# Facilitating Joint Chaos and Fractal Analysis of Biosignals through Nonlinear Adaptive Filtering

**DOI:** 10.1371/journal.pone.0024331

**Published:** 2011-09-06

**Authors:** Jianbo Gao, Jing Hu, Wen-wen Tung

**Affiliations:** 1 PMB Intelligence LLC, West Lafayette, Indiana, United States of America; 2 Department of Mechanical and Materials Engineering, Wright State University, Dayton, Ohio, United States of America; 3 Affymetrix, Inc., Santa Clara, California, United States of America; 4 Department of Earth and Atmospheric Sciences, Purdue University, West Lafayette, Indiana, United States of America; University of Maribor, Slovenia

## Abstract

**Background:**

Chaos and random fractal theories are among the most important for fully characterizing nonlinear dynamics of complicated multiscale biosignals. Chaos analysis requires that signals be relatively noise-free and stationary, while fractal analysis demands signals to be non-rhythmic and scale-free.

**Methodology/Principal Findings:**

To facilitate joint chaos and fractal analysis of biosignals, we present an adaptive algorithm, which: (1) can readily remove nonstationarities from the signal, (2) can more effectively reduce noise in the signals than linear filters, wavelet denoising, and chaos-based noise reduction techniques; (3) can readily decompose a multiscale biosignal into a series of intrinsically bandlimited functions; and (4) offers a new formulation of fractal and multifractal analysis that is better than existing methods when a biosignal contains a strong oscillatory component.

**Conclusions:**

The presented approach is a valuable, versatile tool for the analysis of various types of biological signals. Its effectiveness is demonstrated by offering new important insights into brainwave dynamics and the very high accuracy in automatically detecting epileptic seizures from EEG signals.

## Introduction

Biological signals often exhibit both ordered and disordered behavior. Two of the most important theories for biosignal analysis are chaos theory and random fractal theory [1], [2]. Chaos theory is mainly concerned about apparently irregular behaviors in a complex system that are generated by nonlinear deterministic interactions with only a few degrees of freedom, where noise or intrinsic randomness does not play an important role. For it to be applicable, signals under study have to come from a predominately deterministic system, be relatively noise free, and be stationary (i.e., the statistics of the signals remain fairly constant over time). For a better understanding of the concepts of stationarity, contamination with noise, and determinism, we refer to [3]–[5] for some simple and very illustrative examples. On the other hand, random fractal theory assumes that the dynamics of the system are inherently random and requires the signals be scale-free. Therefore, the foundations of chaos theory and random fractal theory are fundamentally different.

Experimental biological signals are often noisy and nonstationary. These factors complicate tremendously analysis of biosignals using chaos theory. On the other hand, fractal analysis may be hindered by rhythmic activity, which is a signature of biology but is incompatible with the notion of scale-free. These problems can at best partially be mitigated by frequency-domain filtering or wavelet analysis. Rapid accumulation of complex data in life sciences has made it increasingly important to develop new methods to better cope with these difficulties. Here, we present an adaptive algorithm, which has a number of interesting properties: (1) it can readily remove nonstationarities from the signal, including baseline drifts and signal components due to nonphysiological body movements; (2) it can more effectively reduce noise in the signals than linear filters, wavelet denoising, and chaos-based noise reduction schemes; (3) it can readily decompose a multiscale biosignal into a series of intrinsically bandlimited functions; (4) it offers a new formulation of fractal and multifractal analysis, and is better than existing methods when a biosignal contains a strong oscillatory component.

## Methods

### 1. Nonlinear adaptive multiscale decomposition

The proposed adaptive algorithm first partitions a time series into segments (or windows) of length 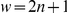 points, where neighboring segments overlap by 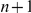 points, and thus introducing a time scale of 
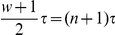
, where 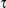 is the sampling time. For each segment, we fit a best polynomial of order 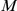. Note that 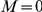 and 1 correspond to piece-wise constant and linear fitting, respectively. Denote the fitted polynomial for the 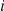-th and 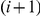-th segments by 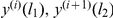, 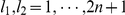, respectively. Note the length of the last segment may be smaller than 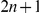. We define the fitting for the overlapped region as
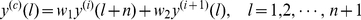
(1)where 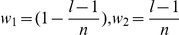 can be written as 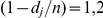, where 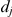 denotes the distances between the point and the centers of 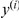 and 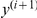, respectively. This means the weights decrease linearly with the distance between the point and the center of the segment. Such a weighting ensures symmetry and effectively eliminates any jumps or discontinuities around the boundaries of neighboring segments. In fact, the scheme ensures that the fitting is continuous everywhere, is smooth at the non-boundary points, and has the right- and left-derivatives at the boundary.

**Figure 1:**
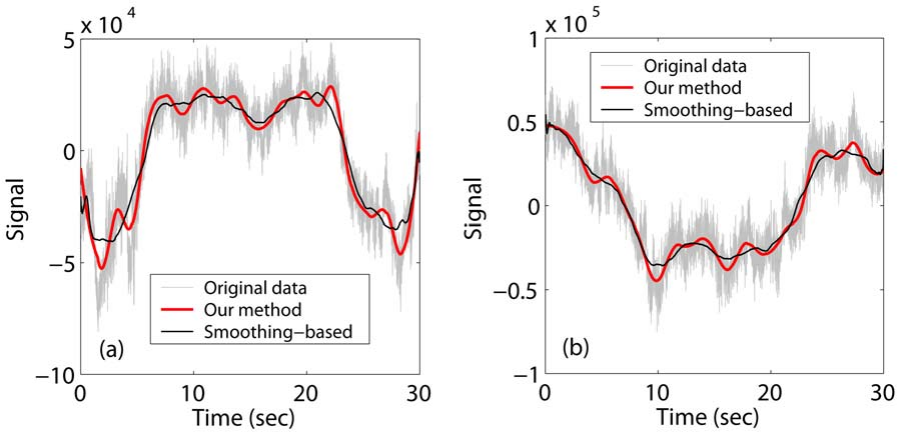
EEG signals with trends removed by the adaptive (thick red) and smoothing-based (thin black) methods.

To appreciate how the algorithm copes with an arbitrary trend without any a-priori knowledge, we have shown in Fig. 1 two scalp EEG signals that were heavily contaminated by head movements. The thick red curves were obtained by the adaptive algorithm, which captured the head movement very well. The thin black curves were obtained by a popular smoothing method based on LOESS [6], which is also a polynomial based nonlinear filtering. Its parameters were chosen to match those of the adaptive filter. While it is also good, it is not as effective.

Since the adaptive detrending can deal with an arbitrary trend without a-priori knowledge, we can conclude that it can readily deal with nonstationarity in a biosignal, including baseline drifts and motion artifacts such as those shown in Fig. 1.

Note that the trend is not necessarily the undesired signal. When it is treated as noise, the adaptive filter is high-pass. When it is considered as signals, the filter is low-pass. When we use two window sizes and take the difference between the trend signals, the filter is band-pass. More generally, if we introduce a series of window sizes, 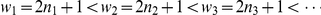, then we get a sequence of trend signals. The difference between two trend signals of window sizes 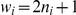 and 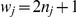 is a band limited signal, with cutoff frequencies 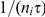 and 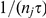, where 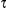 is the sampling time. For convenience, those signals may be called *intrinsically band limited functions (IBFs)* and the procedure multiscale decomposition. This procedure will be made more concrete when we consider fractal structure of sunspot numbers and discuss epileptic seizure detection from EEG in Section *Results*.

**Figure 2:**
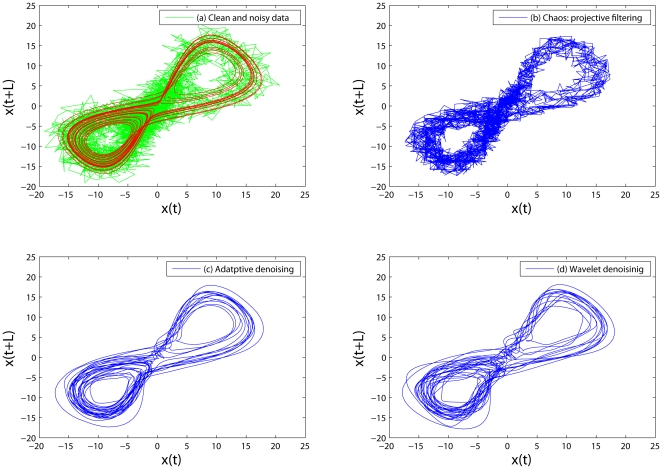
A comparison of proposed adaptive algorithm with wavelet denoising and chaos-based projective filtering for reducing noise in the chaotic Lorenz data. Phase diagrams (i.e., 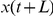 vs. 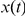) for (a) the clean (red) and noisy (green) Lorenz signal, (b) the signal processed by chaos-based projective filtering, (c) the signal filtered by the proposed adaptive algorithm, and (d) the signal filtered by wavelet denoising.

**Figure 3:**
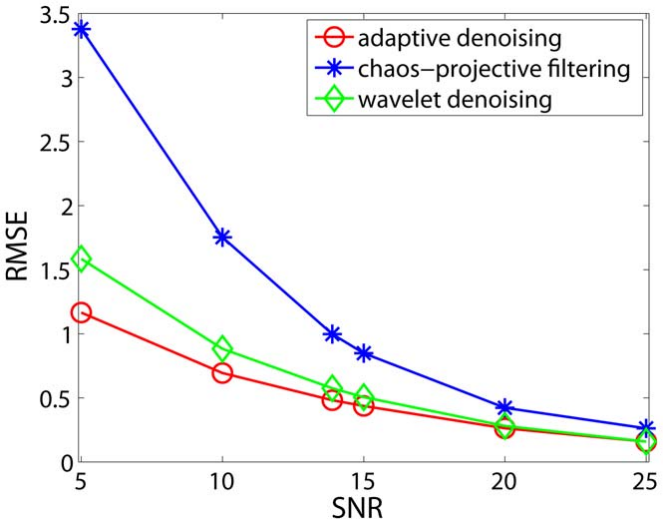
Root Mean Square Error (RMSE) vs. Signal-to-Noise Ratio (SNR) curves for three types of filters (adapted from [**7**]).

In [7], [8], we have shown that the adaptive filter is more effective in reducing noise from time series data than linear filters, wavelet shrinkage, and chaos-based noise reduction schemes. To appreciate this property, we have shown in Fig. 2 a comparison of this algorithm with wavelet denoising and chaos-based projective filtering for reducing noise in the chaotic Lorenz data. Indeed, we observe that the adaptive denoising is the most effective. This can be further corroborated by the smallness of the remaining noise, the root mean square error (RMSE), shown in Fig. 3.

### 2. Fractal and multifractal analysis based on adaptive multiscale decomposition


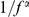
 noise, a form of temporal or spatial fluctuation characterized by a power-law decaying power spectral density, has been observed in numerous natural and man-made systems [9]–[22]. Of particular interest is to understand the correlation structure of such processes, which is characterized by the Hurst parameter 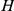, which is equal to 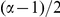 or 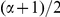 depending on whether the process is a random walk process or a noise (i.e., increment) process – the process is said to have anti-persistent, short-range, or persistent long-range correlations when 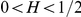, 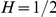, and 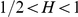, respectively [1], [23].

To better understand the meaning of 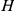, it is useful to mathematically be more precise. Let {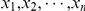} be a stationary stochastic process with mean 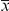 and autocorrelation function of the type,
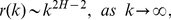
(2)This is often called an increment (or noise) process. Its power spectral density (PSD) is 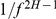. Its integration,
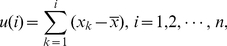
(3)is called a random walk process having PSD 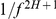. Simple non-overlapping smoothing of {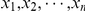} yields a new time series,
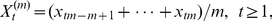
(4)with variance
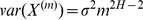
(5)where 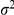 is the variance of original stochastic process {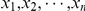}. Eq. (5) offers an excellent means of understanding 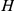. For example, if 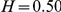, 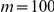, then 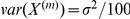. When 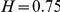, in order to have 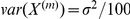, then we need 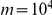, which is much larger than 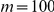 for the case of 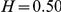. On the other hand, when 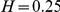, if we still want 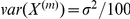, then 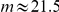, much smaller than 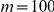, the case of 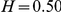. An interesting lesson from such a simple discussion is that if a time series is short while its 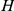 is close to 1, then smoothing is not a viable option for reducing the variations there.

Many excellent methods have been proposed for estimating 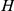. The most popular is perhaps the detrended fluctuation analysis (DFA) [24]. Indeed, it is among the most reliable [23]. The adaptive decomposition algorithm proposed here can be used to formulate a new fractal and multifractal analysis approach, and is even better than DFA when a signal contains a strong trend. For convenience, we call it AFA.

AFA works as follows. If we start from an increment process, 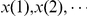, similar to DFA, we first construct a random walk process using Eq. (3). If the original data can be considered as a random walk-like process, which is true for EEG [1], [25], [26] and sea clutter radar returns [23], [27], [28], then this step is not necessary. However, for ideal fractal processes, there is no penalty if this is done, even though the process is already a random walk process.

Next, for a window size 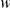, we determine, for the random walk process 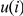 (or the original process if it is already a random walk process), a global trend 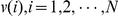. Here 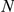 is the length of the random walk process. The residual, 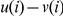, characterizes fluctuations around the global trend, and its variance yields the Hurst parameter 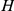,
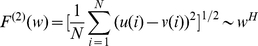
(6)To prove Eq. (6), we start from an increment process with 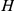. The PSD for the corresponding random walk process, is 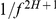. Using Parseval's theorem [1], The variance of the residual data corresponding to a window size 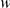 may be equated to the total power in the frequency range (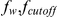),
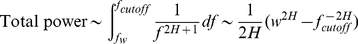
(7)where 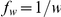, and 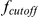 is the highest frequency of the data. When 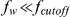, we immediately see that Eq. (6) has to be valid. In fact, the above proof makes it clear that even if we start from a random walk process with 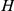, integration will make the process to have a spectrum of 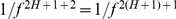, and therefore, the final “Hurst” parameter will be simply 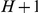. This indicates that there is no penalty if one uses Eq. (3) when the data are already a random walk process.

To extend Eg. (6) to a multifractal formulation, we can simply write
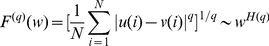
(8)where 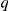 is a real number: depending on whether 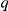 is positive or negative, large or small values of deviations are emphasized, respectively. In many applications, the case of 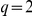 may be most concerned, since 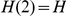. For notational convenience, 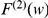 may be simply denoted as 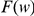.

Eq. (6) can also be extended to high-dimensional case, such as an image or a high-dimensional trajectory. In the case of 2-D, this can be achieved by first applying the algorithm to the 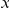-component of the data, then applying it to the 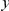-component. In fact, the order of whether 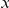-component first or 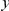-component first does not matter. This is best seen by considering polynomial order to be 1 and functions 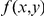 having the property 
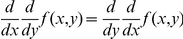
. The approach will work in more general situations, including non-differentiable random surfaces.

The fractal analysis approach formulated here has two important features that are better than DFA: (1) the trend for each window size 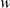 obtained here is smooth, while that obtained by DFA changes abruptly at the boundary of neighboring segments; (2) it can more readily estimate 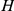 from a signal with a strong oscillatory trend. The latter property will be made clearer when we analyze the sunspot numbers in Section *Results*.

## Results

### 1. Analysis of sunspot numbers

**Figure 4:**
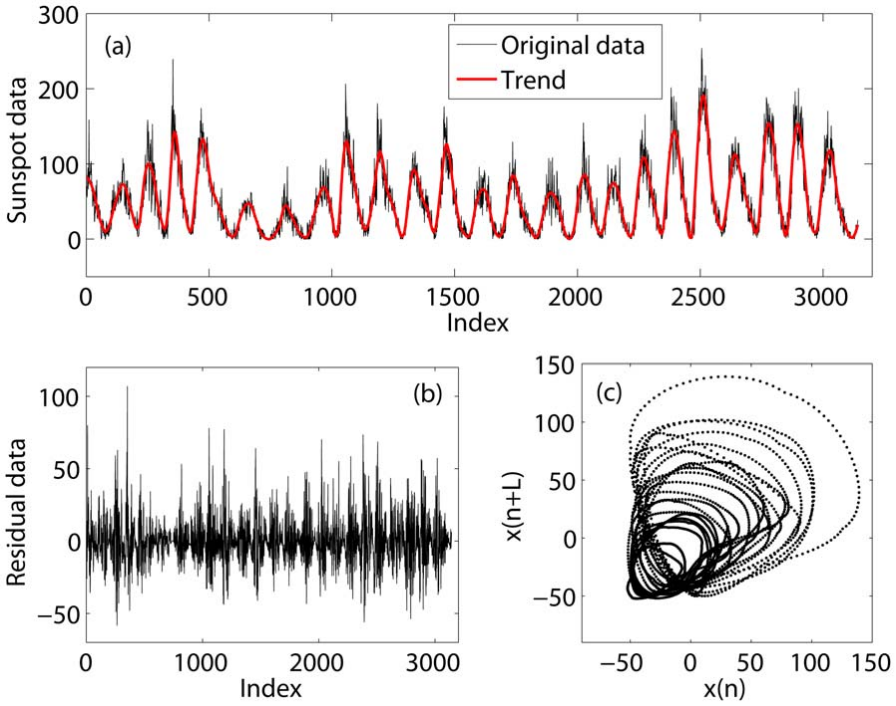
Sunspot data and its trend. (a) original sunspot data (black curve) and the trend data (thick red curve) obtained by adaptive detrending algorithm with a window size of 61 month and a polynomial order of 2; (b) the corresponding residual data; (c) 2-D phase diagram, 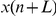 vs. 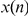 with 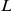 = 30, for the trend data (with mean removed).

To appreciate the effectiveness of AFA, we examine how it estimates the fractal scaling exponent from sunspot numbers (which can be downloaded at http://sidc.oma.be/sunspot-data/). The best known property of sunspot numbers is the approximate 11 year cycle, which can be clearly seen from the data shown in Fig. 4(a). Because of this cyclic trend, DFA cannot readily detect the fractal structure in the sunspot variations [29].

Sunspot numbers up to year 2006 have been examined by Movahed et al. [30] using Fourier filtering based DFA, by Zhou and Leung [31] using empirical mode decomposition (EMD) based DFA, and by Hu et. al. [29] by first using the adaptive detrending algorithm described here then applying DFA. The results based on EMD is consistent with that of Hu et. al. [29]. The latter is much simpler. Referring to Fig. 4, the latter approach is to first get the trend data, shown as the solid black curve in Fig. 4(a) (whose phase diagram is shown in Fig. 4(c), which suggests chaos-like dynamics), then obtains the residual signal shown in Fig. 4(b), and finally applies DFA to the residual signal. The 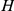 parameter for the shorter data analyzed in Hu et. al. [29] is about 0.74. When the same approach is applied to the longer data analyzed here, 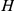 is 0.78. Therefore, the variation of the sunspot numbers around its 11-year cycle is a fractal process with long-range correlations.

**Figure 5:**
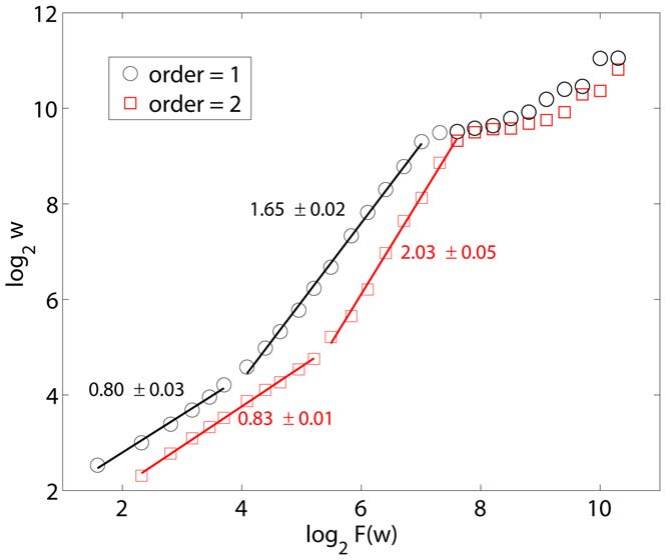
Adaptive fractal analysis of sunspot numbers with polynomial order 1 and 2.

When we apply AFA to the sunspot numbers, we obtain the results shown in Fig. 5. 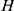 estimated with polynomial order 1 is 0.80, with a short scaling range, while that estimated with polynomial order 2 is 0.83, with a fairly long fractal scaling range up to about 60 months, or half of the 11-year cycle. Therefore, 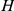 value estimated is consistent with that by other more complicated methods, including EMD based DFA and adaptive detrending based DFA.

### 2. Epileptic seizure detection from EEG

We now demonstrate how AFA can shed new lights on the dynamics of brainwaves and help detect epileptic seizures from EEG.

**Figure 6:**
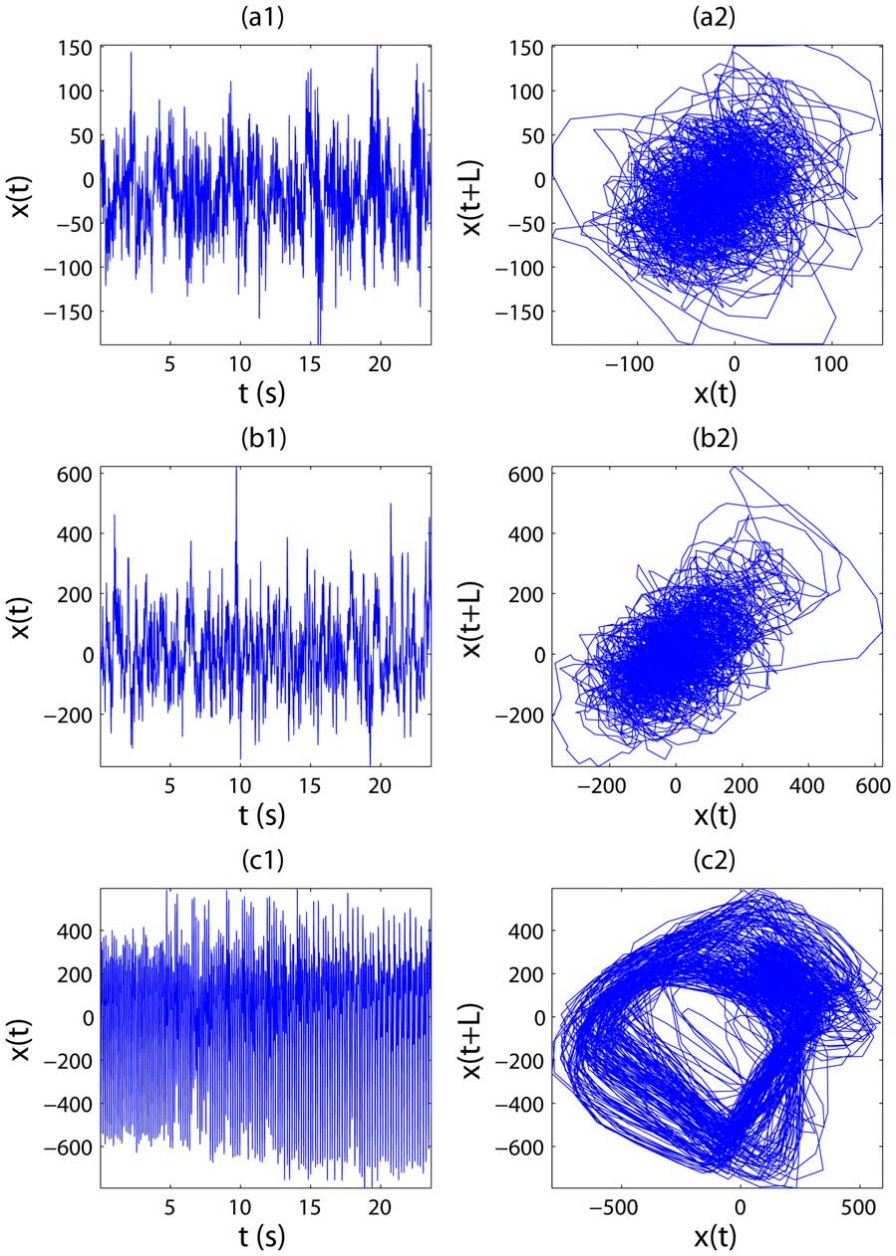
Examples of different groups of EEG signals and corresponding phase diagrams. EEG signals for (a1) H (healthy); (b1) E (epileptic subjects during a seizure-free interval) and (c1) S (epileptic subjects during seizure); (a2,b2,c2) are their corresponding phase diagrams.

Following earlier studies, we treat EEG as a random walk process instead of increment process, therefore, the first step, forming a random walk process, is not necessary here. For ease of comparison with the result of [32], we shall work on the same data sets analyzed there, which consist of three groups, H (healthy), E (epileptic subjects during a seizure-free interval), and S (epileptic subjects during seizure), each group contains 100 data segments, whose length is 4097 data points with a sampling frequency of 173.61 Hz. The data can be downloaded at http://www.meb.uni-bonn.de/epileptologie/science/physik/eegdata.html. Examples of the EEG signals for the three groups, H, E, and S, are shown in Figs. 6(a1,b1,c1), together with their phase diagrams in Figs. 6 (a2,b2,c2). For the details of the data, we refer to [33].

**Figure 7:**
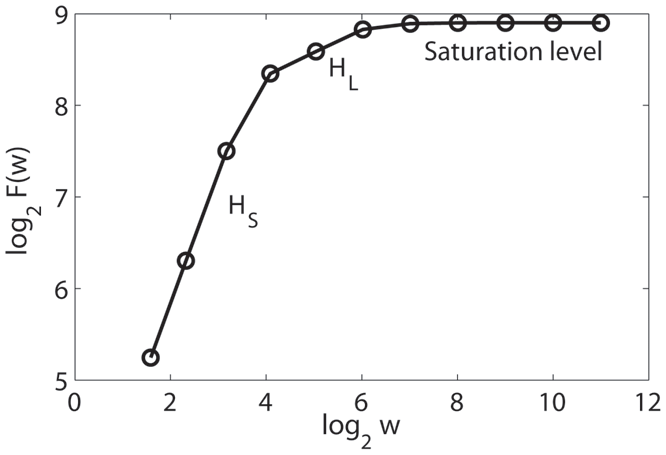
A typical 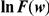 vs. 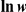 curve for an EEG signal.

**Figure 8:**
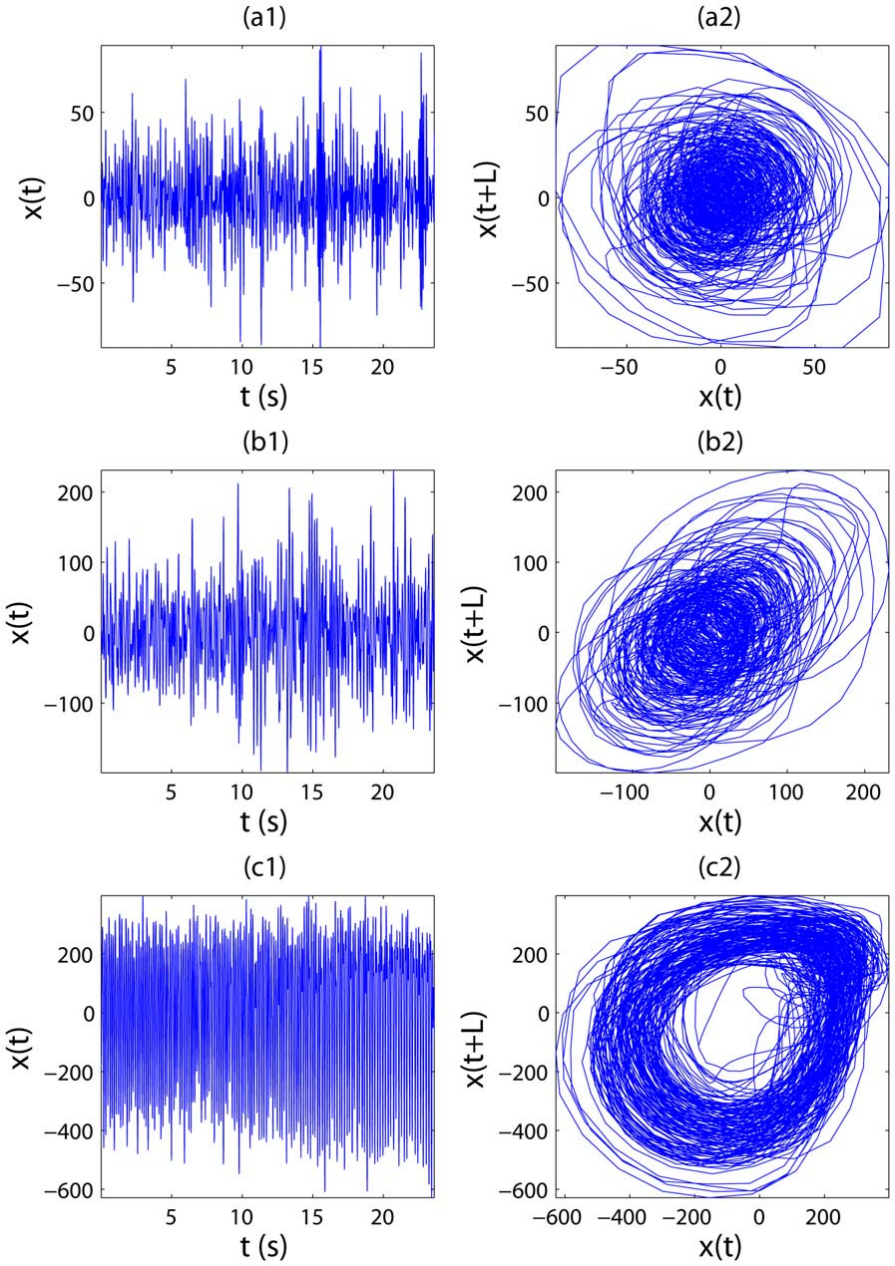
Intrinsically bandlimited functions (IBFs) and phase diagrams for different groups of EEG signals. (a1,b1,c1) are IBFs of EEG signals shown in Fig. 6; (a2,b2,c2) are their corresponding phase diagrams.

Figure 7 shows a typical 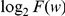 vs. 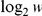 curve for an EEG signal. We observe that there are two short scaling regions, whose Hurst parameters are denoted as 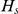 and 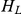 in the plot. The first scaling determines a time scale of 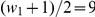 samples, which amounts to 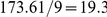 Hz. The second scaling break determines a time scale of 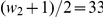 samples, which amounts to 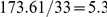 Hz. Using these two time scales, we can obtain two trend signals for each EEG signal. Their difference yields one IBF for each EEG signal. They are shown in Figs. 8(a1,b1,c1) for the signals of Figs. 6(a1,b1,c1). The corresponding phase diagrams are shown in Figs. 8(a2,b2,c2). Fig. 8(c2) is especially interesting, since it suggests chaos-like dynamics for the seizure EEG signal.

**Figure 9:**
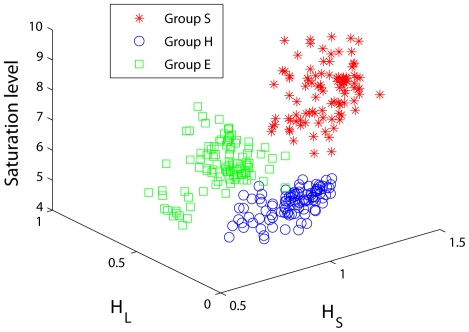
Epileptic seizure detection using the three features derived from adaptive fractal analysis.

When we use the three parameters, 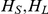, and the saturation level to classify the three EEG groups, we obtain the results shown in Fig. 9. We observe that the three groups almost perfectly separate. This excellent classification result suggests that the two time scales identified above must be generic. This is indeed so, after we visually examine a large subset of the data analyzed here. Note that such an excellent classification accuracy cannot be obtained by using DFA.

It is interesting to note that the seizure detection accuracy shown in Fig. 9 is comparable to that of Adeli et. al. [32] using a complicated approach consisting of (1) decomposing the EEG signals into delta, theta, alpha, beta, and gamma subbands, (2) calculating features such as standard deviations, largest Lyapunov exponent, and correlation dimension for each subband, and (3) using neural networks to classify the three different EEG groups. In particular, it is noted [32] that correlation dimension is more useful for the beta (13–30 Hz) and gamma (30–60 Hz) subbands, while the Lyapunov exponent is more useful for the alpha (8–12 Hz) band. While qualitatively, this observation is consistent with our finding [34] that the largest Lyapunov exponent, because of the particular algorithm of computing it, is more pertinent to larger scale (i.e., slower or lower-frequency dynamics), while correlation dimension characterizes smaller scale (i.e., faster or higher-frequency dynamics). AFA presented here has suggested that the more precise time scales are not given by the traditional idea of the 5 EEG subbands, but are given by the fractal scaling breaks, which are 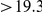 Hz and 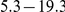 Hz.

## Discussion

Motivated by the pressing need of joint chaos and fractal analysis of complex biological signals, we have proposed a nonlinear adaptive algorithm, which has a number of interesting properties, including removing arbitrary nonphysiological trends or baseline drifts from physiological data, reducing noise, and carrying out fractal analysis. The latter property is utilized to analyze sunspot numbers and three different EEG groups for the purpose of detecting epileptic seizures. It is found that the approach is highly effective. In particular, we have found that the approach can automatically partition the frequency into three bands, below 5.3 Hz, above 19.3 Hz, and between 5.3 and 19.3 Hz. This suggests that a more convenient and more intrinsic way of partitioning EEG signals would be to partition them into these three bands, instead of the traditional delta, theta, alpha, beta, and gamma subbands.

The validity of the proposed approach hinges on being able to locally represent a continuous time function by its Taylor series. Therefore, it will work better when the signal is sampled more densely. This is especially true when denoising is concerned. On the other hand, it may lose power when dealing with signals generated by discrete maps or sampled from a continuous time system with very large sampling time. We do not expect this to be a true difficulty, however, since experimental systems usually are continuous time systems, and there is no shortage of technology to adequately sample the dynamics of the system.

While we have used sunspot numbers and EEGs for example applications, we surmise that the approach proposed here can readily be used to analyze a broad range of biological and non-biological signals. Furthermore, some of the IBFs (such as shown in Fig. 4(c) and Fig. 8(c2)) may better be amenable to chaos analysis. To maximally realize the potential of the approach, interested readers are welcome to contact the authors for the codes.
